# Factors Associated with Depressive Symptoms among Filipino University Students

**DOI:** 10.1371/journal.pone.0079825

**Published:** 2013-11-06

**Authors:** Romeo B. Lee, Madelene Sta. Maria, Susana Estanislao, Cristina Rodriguez

**Affiliations:** 1 Department of Behavioral Sciences, de la Salle University, Manila, The Philippines; 2 Department of Psychology, de la Salle University, Manila, The Philippines; 3 Office of Counselling and Career Services, de la Salle University, Manila, The Philippines; Iran University of Medical Sciences, Islamic Republic of Iran

## Abstract

Depression can be prevented if its symptoms are addressed early and effectively. Prevention against depression among university students is rare in the Philippines, but is urgent because of the rising rates of suicide among the group. Evidence is needed to systematically identify and assist students with higher levels of depressive symptoms. We carried out a survey to determine the social and demographic factors associated with higher levels of depressive symptoms among 2,436 Filipino university students. The University Students Depression Inventory with measures on lethargy, cognition-emotion, and academic motivation, was used. Six of the 11 factors analyzed were found to be statistically significantly associated with more intense levels of depressive symptoms. These factors were: frequency of smoking, frequency of drinking, not living with biological parents, dissatisfaction with one’s financial condition, level of closeness with parents, and level of closeness with peers. Sex, age category, course category, year level and religion were not significantly related. In identifying students with greater risk for depression, characteristics related to lifestyle, financial condition, parents and peers are crucial. There is a need to carry out more surveys to develop the pool of local knowledge on student depression.

## Introduction

Depression is a major source of the burden of disease throughout the world [1]. In much of the developing world, however, depression is largely unexplored as a research topic. A social mapping revealed that, even though the mental disorder has been recognized as a research priority, only a sparse number of relevant studies have been carried out in low- and middle-income countries [2]. Roughly 60% of these countries have contributed fewer than five articles to the international mental health indexed literature [2]. Strategic evidence is needed in order to prevent the occurrence of depression, including its pernicious effects and prohibitive treatment cost. 

Prevention of depression, particularly among university students in developing countries, is urgent. With their large student populations and the developmental propensity of students for depression [3], the burden of the mental disorder is heavy on this demographic sector [4–6]. Preventive efforts in the developing world, however, are rare. Consistent with observations elsewhere [7,8], depression is widely perceived in this part of the world as innocuous and as part and parcel of normal adolescent development. Students with the mental disorder are not only suffering in silence, but are also placing their academic and future life goals in peril. Depression can be averted if students with depressive symptoms, comprising not only physical but also non-physical conditions (e.g., cognition-emotion and motivation) [9], are promptly and properly identified and helped.

Extant studies suggest that students with higher levels of symptoms tend to be women [10,11], older and in their senior year [5], and Catholics and/or Jews [12,13]. Moreover, research indicates that highly symptomatic students do not reside with their parents in one household [14], and are smoking [15] and drinking alcohol [16], and belong to the low-income bracket [6]. Furthermore, students with more severe levels of depressive symptoms have lower levels of closeness with their parents or with friends [7]. 

### The context of the present study

The Philippines has a total population of 92.3 million that is very young (median age: 23) and growing at 1.9% annually. In 2009-2010, 2.8 million university students were enrolled in the country’s 2,247 higher education institutions. Of every 10 Filipino students, 6 and 4 are enrolled in private and public universities, respectively. Of these students, 26% are enrolled in business, 16% in medicine and allied programs, and 13% each are in engineering, information science and education [17]. In contrast to their counterparts throughout most of the world, Filipino students commence their university education at the age of 15 or 16 years.

Filipinos place a high premium on formal education; a university degree is strongly regarded as a primary requirement for social and economic mobility. In the context of the collective aspirations of Filipinos to go abroad for lucrative employments, the need for university education is even more compelling. Individual students are thus pressured to excel or complete a degree, lest they bring dishonor to their family and friends, and endanger their employment and life prospects. In this respect, academic-related matters are salient issues for individual students and in their relationships and conflicts with parents; these, too, can induce higher levels of depressive symptoms in students. 

We carried out this research as part of our community engagement activities to help in the prevention of mental disorders, and subsequently, of suicide among Filipino university students. The connection between depression and suicide is well-established [18]. The spate of suicide events among local students had served as the impetus to conceive and implement this study. There is paucity of data on university student depressive symptomatology in the Philippines, and in the absence of published relevant articles in indexed journals, little is understood about depressive symptoms among Filipino university students at the international level. This survey examined the social and demographic factors associated with higher levels of depressive symptoms among Filipino university students. The University Student Depression Inventory (USDI), a newly-developed and psychometrically sound scale with measures on academic motivation in addition to lethargy and cognition-emotion, was used. 

## Methods

### Participants

Data were derived from a complete enumeration survey undertaken in 2012 covering all 67 undergraduate classes in general social sciences (e.g., introductory sociology) at a large private university (total student population: >16,000) in Manila, the Philippines. Roughly half of the 67 classes were surveyed in the middle of Term 1 and the other half in the middle of Term 2. A total of 2,591 Filipino students anonymously completed the 10-page self-accomplished questionnaire. Only the questionnaires of 2,436 students were considered for the purpose of this report (126 questionnaires of international students were excluded and 29 questionnaires with at least 10 unanswered items were invalidated). Our sample represents about 15% of the university’s total undergraduate student population. 

### Measures

We utilized the USDI to measure depressive symptoms as a continuous variable. The USDI, developed by Khawaja & Kelly [9], measures the academic motivational aspect of depressive symptoms in addition to physical and cognitive-emotive dimensions. The USDI has 3 sub-scales having a total of 30 statements: lethargy (9 statements on lethargy, concentration difficulties and task performance); cognitive-emotional (14 statements on suicide ideation, worthlessness, emotional emptiness and sadness); and academic motivation (7 statements on class attendance and motivation to study) (Table 1). Statements have score-bearing response options ranging from “none at all” (1) to “all the time” (5). The USDI has a high level of internal consistency (Cronbach α=0.95) [9]. 

**Table 1:** Statements used for measuring levels of closeness with parents and with peers, and depressive symptoms.

Parents
1. I like spending time with my parents.
2. My parent/s show/s how much she/she/they love me.
3. I feel good being with my parents.
4. My parent/s does/do not really care about me.*
5. I disclose my private concerns to my parent/s.
6. I am not happy when I spend time with my parent/s.*
7. I think my parent/s is/are the best in the world.
8. I wish my parent/s paid more attention to me.*
Peers
1. I feel happy when I am with my friends.
2. I would rather be alone than spend time with my friends.*
3. My friends show me their support.
4. My friends do not treat me well.*
5. I wish I had more supportive friends.*
6. I am satisfied with the friendships I develop in school.
7. I like spending time with my friends.
8. I do not enjoy spending time with my friends.*
9. I am happiest when I am with my friends.
Depressive symptoms
A. Lethargy
1. I am more tired than I used to be.
2. I do not have the energy to study at my usual level.
3. My energy is low.
4. I find it hard to concentrate.
5. I don't feel rested even after sleeping.
6. I am overwhelmed by the challenges I encounter in my studies.
7. My mood affects my ability to carry out assigned tasks.
8. Daily tasks take me longer than they used to.
9. My study is disrupted by distracting thoughts.
B. Cognitive/emotional
10. I wonder whether life is worth living.
11. I feel worthless.
12. I have thought about killing myself.
13. No one cares about me.
14. I feel emotionally empty.
15. I feel sad.
16. I worry I will not amount to anything.
17. The activities I used to enjoy no longer interest me.
18. I feel like I cannot control my emotions.
19. I spend more time alone than I used to.
20. I feel disappointed in myself.
21. I feel withdrawn when I'm around with others.
22. I do not cope well.
23. I think most people are better than me.
Academic motivation
24. I do not have any desire to go to my classes.
25. I do not attend classes as much as I used to.
26. I don't feel motivated to study.
27. Going to university is pointless.
28. I have trouble starting assignments.
29. I do not find study as interesting as I used to.
30. I have trouble completing study tasks.
*Reverse coded

The socio-demographic characteristics include sex, age category, course category, year level, religion, frequency of smoking, frequency of drinking, living/not living with both biological parents, level of satisfaction with one’s financial condition, level of closeness with parents, and level of closeness with peers. The last 2 variables were measured using a series of 8 statements on parents and 9 statements on peers. The statements were drawn from published studies on parental and peer relationships among adolescents [7,19]. Each series had 4 score-bearing response options: definitely not true (1), mostly not true (2), mostly true (3), and definitely true (4) (Table 1). 

### Ethical standards

The study was approved by the ethics review committee of the university. After evaluating the contents of the survey instrument, the Committee assessed that the study would have no known risk to research participants. Verbal consent was thus obtained; however, students were informed that they could decline participation and that they could stop completing the questionnaire if they wished to. The benefits of the study (i.e., findings would be used to draw attention towards mental health in Filipino students) were especially stressed in order to trigger a sense of social responsibility and citizenship, and therefore, research participation among students. These instructions were written on the cover page of the survey instrument that was administered. On the same cover page, we also included our full names and contact numbers in which we enjoined students to ask us questions about the study and related matters. 

We did not seek the consent of the students’ parents anymore. The survey focused on real-life conditions (e.g., feeling bored and having low energy) which are normally shared between and among Filipino students. During our pre-test of the questionnaire, student-respondents perceived the topic of the study as personally acceptable, one they felt they would not be asking their parents for permission should they decide to discuss it. The foregoing ethical standards, especially with respect to studies with no known harmful risks and the waiving of a signed certification of consent, are in line with the practices of most Institutional Review Boards elsewhere. 

### Procedure

We conducted the survey in classrooms during the first quarter of the 90-minute classes. Each class was informed about the importance and rationale, and the anonymity and confidentiality of the study. Afterwards, students were invited to participate and were each given a questionnaire to accomplish. Students were reminded not to write any mark in the instrument that would identify them. Whether completely accomplished or not, all questionnaires were collected. Students were thanked for their participation. No incentive of any form was given. 

### Analysis

Using the Statistical Package for the Social Sciences Version 20, differences in the mean depressive symptoms scores were examined based on social and demographic characteristics. The characteristics that were statistically significantly related with higher levels of depressive symptoms were further examined at the sub-scale levels. The analysis of variance was used. 

The independent variables, except for sex (male, female), were recoded into variables with 2-3 categories each (Table 2). The levels of closeness with parents and with peers were constructed by adding the scores corresponding to responses given to the series of statements. For level of closeness with parents, the score range is 8 to 32 (low-moderate, 8-23; high, 24-32); and for level of closeness with peers, the range is 9 to 36 (low-moderate, 9-26; high, 27-36). Our analyses revealed a high level of internal consistency for both series (parents: α=0.77; peers α=0.79). 

**Table 2:** Means and standard deviations for depressive symptoms scale scores by social and demographic characteristics.

**Variables**	**Categories**	**N**	**%**	**Means**	**Standard deviation**
Sex	Male	1063	43.6	71.39	19.21
	Female	1373	56.4	71.47	18.18
Age category	<17	1034	42.5	71.43	18.9
	17	724	29.8	71.17	18.23
	>17	674	27.7	71.75	18.71
Course category	Social sciences and humanities	941	39.0	72.14	18.78
	Business, economics and management	714	29.6	70.02	18.15
	Double major and interdisciplinary	198	8.2	70.54	19.73
	Engineering, natural sciences and computer science	561	23.2	72.47	18.57
Year level	1^st^	1731	71.1	71.16	18.69
	2^nd^-4^th^	704	28.9	72.13	18.47
Religion	Catholic	1968	80.9	71.55	18.38
	Non-Catholic/others	466	19.1	71.04	19.69
Frequency of smoking (in days)**	0	2108	86.5	70.84	18.50
	≤10	182	7.5	76.82	18.64
	>10	146	6.0	73.30	19.47
Frequency of drinking (in days)**	≤10	885	36.5	73.29	19.03
	>10	1541	63.5	70.33	18.28
Living with both biological parents*	Yes	1895	77.9	70.99	18.51
	No	539	22.1	72.99	19.03
Level of satisfaction with one's financial condition**	Not satisfied	140	5.8	81.97	20.84
	Somewhat satisfied	583	24.0	77.19	18.84
	Satisfied	1252	51.6	69.19	17.43
	Very satisfied	452	18.6	66.78	17.86
Level of closeness with parents**	Low/moderate	427	17.5	81.65	19.77
	High	2006	82.5	69.2	17.65
Level of closeness with peers**	Low/moderate	289	11.9	84.58	20.69
	High	2138	88.1	69.66	17.61

* p<.05**p<.01

**Table 3:** Means, F-values and p-values for depressive symptoms sub-scale scores by selected social and demographic characteristics.

**Variables**	**Lethargy**		**Cognition/emotion**		**Academic motivation**	
	**Means**	**p-values**	**Means**	**p-values**	**Mean**	**p-values**
Frequency of smoking (in days)		=0.001**		=0.007**		=0.000**
0	28.42		28.41		14.05	
≤10	30.03		30.76		16.03	
>10	29.51		27.62		16.24	
	F_(2,2432)_=6.56, SS=556.88, MS=278.44		F_(2,2432)_=5.03, SS=1066.29, MS=533.14		F_(2,2432)_=22.82, SS=1222.83, MS=611.41	
Frequency of drinking (in days)		=0.001**		=0.033*		=0.000**
≤10	29.17		29.11		15.11	
>10	28.29		28.18		13.87	
	F_(1,2423)_=10.28, SS=435.77		F_(1,2423)_=4.53, SS=479.96		F_(1,2423)_=32.03, SS=861.29	
Living with both biological parents		=0.098 NS		=0.042*		=0.104 NS
Yes	28.49		28.30		14.24	
No	29.02		29.32		14.65	
	F_(1,2431)_=2.74, SS=116.89		F_(1,2431)_=4.15, SS=441.32		F_(1,2431)_=2.65, SS=72.24, MS=72.24	
Level of satisfaction with one’s financial condition		=0.000**		=0.000**		=0.000**
Not satisfied	31.37		33.69		16.91	
Somewhat satisfied	29.91		31.83		15.46	
Satisfied	28.09		27.40		13.76	
Very satisfied	27.51		25.68		13.59	
	F_(3,2422)_=23.56, SS=2934, MS=978.00		F_(3,2422)_=51.08, SS=15351.53, MS=5117.18		F_(3,2422)_=29.36, SS=2319.56, MS=773.18	
Level of closeness with parents		=0.000**		=0.000**		=0.000**
Low/moderate	30.74		34.35		16.56	
High	28.16		27.29		13.86	
	F_(1,2430)_=56.15, SS=2342.5		F_(1,2430)_=176.73, SS=17563.84		F_(1,2430)_=98.06, SS=2571.45	
Level of closeness with peers		=0.000**		=0.000**		=0.000**
Low/moderate	30.86		37.10		16.91	
High	28.30		27.37		13.98	
	F_(1,2424)_=39.59, SS=1658.98		F_(1,2424)_=248.31, SS=24038.13		F_(1,2424)_=82.43, SS=2178.29	

* p<.05**p<.01. SS=sum of squares. MS=mean squares. NS=not significant

The dependent variable (levels of depressive symptoms) was constructed by adding the scores corresponding to the responses given to the series of statements. The scale score ranges from 30 to 150 while the sub-scale scores range from 9 to 45 for lethargy, 14 to 70 for cognition-emotion, and 7 to 35 for academic motivation; higher scores suggest higher levels of depressive symptoms Our analyses revealed a high level of internal consistency for the USDI (α=0.93). 

## Results

### Profile of respondents

The majority were female while 43.6% were male. 42.5% were 16 years of age or younger, 29.8% were 17 years old and a similar number were older. 39.0% were in social sciences/humanities; 29.6% were in business/economics/management and 23.2% were in engineering/natural/computer sciences. Seven of every 10 were first year students. Most were Catholic (80.9%) and reported not having smoked in the past 30 days prior to the survey. In the past 30 days, about 6 of every 10 students had taken alcohol for more than 10 days, while 4 for ≤10 days. Most respondents (77.9%) currently lived with both biological parents. About 70% were satisfied and very satisfied with their financial condition; the rest were not or were only somewhat satisfied. Most had high levels of closeness with parents (82.5%) and peers (88.1%). 

### Differences in mean scale scores based on social and demographic characteristics

The means and standard deviations for depressive symptoms scale scores are shown in Table 2. Higher means suggest higher or more severe levels of depressive symptoms. Results indicate that male and female students did not differ in their symptoms levels. No significant differences were observed across age groups. The level of depressive symptoms statistically significantly varied according to course category but only marginally (F_(3,2410)_=2.54, p<.06). Means were not significantly dissimilar across year level and religion. 

Means comparison related to frequency of smoking suggests significant differences among the categories (F_(2,2411)_=9.65, p<.01). Results of post-hoc Tukey test indicate that those who smoked for ≤10 days had a higher level of depressive symptoms than those who did not smoke in the past 30 days (p<.01). Significant means differences were observed based on frequency of drinking (F_(1,2424)_=14.31, p<.01). Students not living with both parents had a significantly higher level of symptoms compared to those living with parents (F_(1,2432)_=4.87, p<.05). Moreover, depressive symptoms level significantly varied according to satisfaction with one’s financial condition (F_(3,2423)_=52.03, p<.01). Based on post-hoc Tukey test findings, students who were not satisfied with their financial status had a more elevated level of depressive symptoms than those who were somewhat satisfied (p<.05), satisfied (p<.01) and very satisfied (p<.01). 

Students with a low to a moderate level of closeness with parents had a significantly higher level of depressive symptoms than students with a high level of closeness with parents (F_(1,2431)_=165.76, p<.01). Students with a low-moderate level of closeness with peers had a significantly higher level of symptoms than those with a high level of closeness with peers (F_(1,2425)_=176.91, p<.01). 

The 6 independent variables with statistically significant relationships with higher levels of depressive symptoms were further examined for their interactions. The two-way analysis of variance results indicate an absence of any interaction. 

### Differences in mean sub-scale scores based on statistically significant social and demographic factors

Additional analyses using the one-way analysis of variance were performed to determine if the statistically significant associations of the 6 independent variables (i.e., frequency of smoking, frequency of drinking, living/not living with both biological parents, level of satisfaction with financial condition, level of closeness with parents, and level of closeness with peers) would hold at the sub-scale level. The means, F-values and p-values are given in Table 3. 

Results indicate that the associations of the 5 variables (i.e., frequency of drinking, level of satisfaction with financial condition, and levels of closeness with parents and with peers) persisted at all sub-scales of depressive symptoms (p-values at <0.01 or <0.05). The significant sub-scale association of the remaining variable (i.e., living/not living with both biological parents) was confined only to the cognitive-emotional sub-scale. 

## Discussion

This survey identified a set of social and demographic factors that are statistically significantly associated with higher levels of depressive symptoms among Filipino university students. The aim is to help prevent depression among the domestic university student population. If students with elevated risks are known and assisted early, their depression would be promptly averted. Data suggest that the factors with significant associations with depressive symptoms, mostly at both the scale and sub-scale levels, were frequency of smoking, frequency of drinking, living/not living with both biological parents, level of satisfaction with one’s financial condition, and levels of closeness with parents and with peers. 

The significant associations of frequencies of smoking and of drinking with depressive symptoms are aligned with extant empirical findings [20,21]. The present study revealed that Filipino students who smoked for some days (against those who did not smoke) and who took alcohol for some days (against those who consumed alcohol for longer durations) had higher depressive symptoms levels. In explaining the associations of smoking and drinking, some studies tend to highlight the psychopharmacological [20] and symbiotic [22] dimensions of these bivariate relationships. This implies that students could have smoked or taken alcohol as an escape route from the burdens of psychosocial difficulties. In the case of drinking, in particular, the use of alcohol usually precedes the symptoms of lethargy and social difficulties associated with depression [23,24]. Caution should be taken in appreciating these interpretations, however. The variables were measured in this study based on the number of days of smoking and drinking rather than the quantities of cigarettes and alcohol consumed (these two are not necessarily equivalent indicators). Considering that the rates of smoking and drinking among the Filipino youth are relatively high (21.0% and 41.4%, respectively) [25], these twin behaviors, specifically their frequencies, need closer examination vis-à-vis depressive symptoms. 

The association between not living in the household with both biological parents and having more serious levels of depressive symptoms has ample empirical support [14,26]. Across the country, many Filipino students do not reside with both parents while pursuing their university education, because they live away from home in dormitories and/or their biological parents are single, separated, or are working abroad. Either as a permanent or a temporary condition, not living with both biological parents may induce depressive symptoms, primarily in cognitive-emotive terms as this study revealed, probably as a result of having restricted access to parental presence and support. 

Satisfaction or dissatisfaction with one’s financial condition is well-confirmed in several other investigations for its significant role in mental health [27]. It is usually expensive to study in a private Philippine university compared to studying in the country’s state colleges and universities. Students in private universities would generally belong to higher levels of socioeconomic status and may influence a peer culture that promotes greater awareness of a person’s socioeconomic standing in society. Such an educational environment is, in turn, likely to enhance sensitivities about one’s own social status in comparison to one’s peers. Those who perceive themselves as higher in status also have higher levels of optimism and perceived control, and therefore, are also likely to exhibit lower levels of depressive symptoms [28,29]. 

The current study findings on the significant associations between the levels of closeness with parents and peers and depressive symptoms are to be expected; these are within the realm of the evidence widely reported in other investigations [7,30]. That most of the Filipino university students who participated in this study had high closeness levels with their parents and peers is hardly unexpected. Parents and friends are basic yet very significant primary groups for Filipino adolescents. Their provisions, including the immediate care, security and support that they bestow and the secure attachments that they consequently foster, are effective protectors and buffers of university students against depressive symptoms [31,32].

In the absence of high level of closeness of Filipino students with parents, in which the parent-child relationship would be characterized by communication problems, excessive parental control, low levels of cohesion, and high levels of conflict in the families, adolescents are bound to experience depressive symptoms [33,34]. Without high level of closeness with peers, local students are also predisposed to be at risk. Students are in a stage when they mostly need their peers for emotional support. Peer acceptance is important to the growing individual and is therefore associated with depressive symptoms [35]. Compared to the association of the lack of parental warmth and acceptance with adolescents’ depressive symptoms, which is largely unidirectional, the association between depressive symptoms and peer-relational problems tends to be bidirectional [36]. Filipino students exhibiting depressive symptoms are likely to be spending less time interacting with their peers and are prone to relate with them aggressively. This interaction pattern, in turn, is likely to cultivate further peer rejection and neglect. 

Sex, age category, course category, year level and religion were not statistically significant factors as our analyses revealed. As a general rule, females show higher rates of depression than males [37,38] due to their tendency to be more expressive and more sensitive to the support provided by their social networks [39]. However, this normative rule on gender differences does not seem to hold true for university students [37]. The exception may be accounted for by the homogeneous university life experiences, similarities in parental education, or common socio-demographic conditions among the youth in general [37,39]. The lack of significant associations of age category, course category and year level among Filipino students could be due to this homogeneity factor as well, particularly that most of them were young, freshmen and completing general education rather than major subjects at the time of their interview. Religion was not significantly associated with depressive symptoms and this is to be expected: the Filipino youth, including university students, are largely nominal Catholics who seldom practice their faith [40]. Elsewhere, one’s religiousness rather than religious affiliation per se has been observed to be significantly related with lower levels of depressive symptoms in students [41]. 

The survey has some limitations. Since the study’s respondents were from general education classes with mostly first year students from middle- and high-income backgrounds, the findings cannot be generalized to the entire student population of the university surveyed or student populations from other universities in the Philippines. Another limitation of the survey is that it did not include other factors that may have potential relationships with higher levels of depressive symptoms. For instance, since completing a university degree is culturally valued among Filipinos, the academic performance of students could be a critical factor for assessing depressive symptoms. Also, the study is cross-sectional, and as such, its conclusions only refer to associations rather than causal relationships between the independent and dependent variables. Moreover, the level of depressive symptoms measured through the USDI pertains not to the sequence of the occurrence of high levels of depressive symptoms, but to the amount of depressive symptoms weighted by frequency of occurrence students experienced in the past fortnight. 

More surveys using the USDI are needed in the Philippines. Future studies have to involve representative samples of Filipino university students from other socio-economic backgrounds. If feasible, longitudinal studies, which will provide repeated observations of the levels and associated factors of depressive symptoms, are a better alternative. Variables related to students’ academic performance should be included as well. Some variable measures (e.g., frequency of smoking) need to capture more nuanced dimensions of the social and demographic conditions of students at greater risk, For example, variables related to smoking and drinking should ask follow-up questions regarding the specific quantities of cigarettes and alcohol consumed by students in a given period. In addition, the association of religion with depressive symptoms will be better understood by a follow-up question on religiousness. 

## Conclusion

The present survey is a pioneering large-scale research on the social and demographic factors of higher levels of depressive symptoms among Filipino university students. These initial findings can help guide the development of a campus-based prevention program at the university surveyed. Towards addressing depressive symptoms and depression in students, lifestyle and factors related to financial condition and parental and peer relationships are important considerations for identifying those at greater risk. More research is needed towards building additional local knowledge on the topic. 
